# Social media discourse and internet search queries on cannabis as a medicine: A systematic scoping review

**DOI:** 10.1371/journal.pone.0269143

**Published:** 2023-01-20

**Authors:** Christine Mary Hallinan, Sedigheh Khademi Habibabadi, Mike Conway, Yvonne Ann Bonomo

**Affiliations:** 1 Faculty of Medicine, Department of General Practice, Dentistry & Health Sciences, The University of Melbourne, Melbourne, Victoria, Australia; 2 Faculty of Medicine, Department of General Practice, Health & Biomedical Research Information Technology Unit (HaBIC R2), Melbourne Medical School, Dentistry & Health Sciences, The University of Melbourne, Melbourne, Victoria, Australia; 3 Centre for Digital Transformation of Health, Victorian Comprehensive Cancer Centre, The University of Melbourne, Melbourne, Victoria, Australia; 4 St Vincent’s Health—Department of Addiction Medicine, Melbourne, Victoria, Australia; 5 Faculty of Medicine, St Vincent’s Clinical School, Melbourne Medical School, Dentistry & Health Sciences, The University of Melbourne, Melbourne, Victoria, Australia; University of Toronto, CANADA

## Abstract

The use of cannabis for medicinal purposes has increased globally over the past decade since patient access to medicinal cannabis has been legislated across jurisdictions in Europe, the United Kingdom, the United States, Canada, and Australia. Yet, evidence relating to the effect of medical cannabis on the management of symptoms for a suite of conditions is only just emerging. Although there is considerable engagement from many stakeholders to add to the evidence base through randomized controlled trials, many gaps in the literature remain. Data from real-world and patient reported sources can provide opportunities to address this evidence deficit. This real-world data can be captured from a variety of sources such as found in routinely collected health care and health services records that include but are not limited to patient generated data from medical, administrative and claims data, patient reported data from surveys, wearable trackers, patient registries, and social media. In this systematic scoping review, we seek to understand the utility of online user generated text into the use of cannabis as a medicine. In this scoping review, we aimed to systematically search published literature to examine the extent, range, and nature of research that utilises user-generated content to examine to cannabis as a medicine. The objective of this methodological review is to synthesise primary research that uses social media discourse and internet search engine queries to answer the following questions: (i) In what way, is online user-generated text used as a data source in the investigation of cannabis as a medicine? (ii) What are the aims, data sources, methods, and research themes of studies using online user-generated text to discuss the medicinal use of cannabis. We conducted a manual search of primary research studies which used online user-generated text as a data source using the MEDLINE, Embase, Web of Science, and Scopus databases in October 2022. Editorials, letters, commentaries, surveys, protocols, and book chapters were excluded from the review. Forty-two studies were included in this review, twenty-two studies used manually labelled data, four studies used existing meta-data (Google trends/geo-location data), two studies used data that was manually coded using crowdsourcing services, and two used automated coding supplied by a social media analytics company, fifteen used computational methods for annotating data. Our review reflects a growing interest in the use of user-generated content for public health surveillance. It also demonstrates the need for the development of a systematic approach for evaluating the quality of social media studies and highlights the utility of automatic processing and computational methods (machine learning technologies) for large social media datasets. This systematic scoping review has shown that user-generated content as a data source for studying cannabis as a medicine provides another means to understand how cannabis is perceived and used in the community. As such, it provides another potential ‘tool’ with which to engage in pharmacovigilance of, not only cannabis as a medicine, but also other novel therapeutics as they enter the market.

## Introduction

Genetic analysis of ancients cannabis indicates the plant cannabis sativa was first cultivated for use as a as a medicinal agent up to 2400 years ago [1]. From the 1800’s, people in the United States (US), widely used cannabis as a medicine by either prescription or as an over the counter therapeutic [2]. Yet by the mid-20th century, cannabis use was prohibited in many parts of the developed world with the passing of legislation in the US, the United Kingdom (UK) and various European countries that proscribed its use [3–6]. Since the 2000s, the use of cannabis for medicinal purposes has been decriminalized in many countries including Israel, Canada, Netherlands, United States, United Kingdom, and Australia [7–9]. More recently, new evidence regarding the clinical effect of medical cannabis on the management of symptoms for some conditions [10] has triggered public interest in cannabis and cannabis-derived products [11,12], resulting in a global trend towards public acceptance, and subsequent legalisation of cannabis for both medicinal and non-medicinal use.

There is emerging evidence of cannabis efficacy for childhood epilepsy, spasticity, and neuropathic pain in multiple sclerosis, acquired immunodeficiency syndrome (AIDS) wasting syndrome, and cancer chemotherapy-induced nausea and vomiting [13–15]. Although researchers are investigating cannabis for treating cancer, psychiatric disorders [16], sleep disorders [17], chronic pain [18] and inflammatory conditions such as rheumatoid arthritis [19], there is currently insufficient evidence to support its clinical use. Scientific studies on emerging therapeutics typically exclude vulnerable populations such as pregnant women, young people, the elderly, patients with multimorbidity and polypharmacy, and this limits the availability of evidence for cannabis effectiveness across these population groups [20].

Cannabis as medicine is associated with a rapidly expanding industry [21]. Patient demand is increasing, as is reflected in an increasing number of approvals for prescriptions over time [22], with one study showing that 61% of Australian GPs surveyed reported one or more patient enquiries regarding medical cannabis [23]. With this increasing demand, is sophisticated marketing by medicinal cannabis companies that leverages evidence from a small number of studies to promote their products [24,25]. In light of this, concerns regarding patient safety is warranted especially when marketing for some cannabinoid products is associated with inadequate labelling and/or inappropriate dosage recommendations [26]. These concerns are compounded by the downscheduling of over-the-counter cannabis products which do not require a prescription [27] and the illicit drug market [28]. Given this dynamic interplay between marketing, product innovation, regulation, and consumer demand, innovative methods are required to augment existing established approaches to the surveillance and monitoring of emerging and unapproved drugs.

Although there is considerable engagement from many stakeholders to improve the scientific evidence regarding the efficacy and safety of cannabis through randomised controlled trials, many gaps remain in the literature [29]. Yet data from real-world and patient reported data sources could provide opportunities to address this evidence deficit [30]. This real-world data can be captured from a variety of sources such as found in routinely collected health care and health services records that include but are not limited to patient generated data from medical, administrative and claims data, as well as patient reported data from surveys, wearable trackers, patient registries, and social media [31–33].

People readily consult the internet when looking for and sharing health information [34,35]. According to 2017 survey of Health Information National Trends, almost 78% of US adults used online searches first to inquire about health or medical information [34]. Data resulting from these online activities is labelled ‘user generated’ and is increasingly becoming a component of surveillance systems in the health data domain [36]. Monitoring user-generated data on the web can be a timely and inexpensive way to generated population-level insights [37]. The collective experiences and opinions shared online are an easily accessible wide-ranging data source for tracking emerging trends–which might be unavailable or less noticeable by other surveillance systems.

The objective of this systematic scoping review is to understand the utility of online user generated text in providing insight into the use of cannabis as a medicine. In this review, we aim to systematically search published literature to examine the extent, range, and nature of research that utilises user-generated content to examine cannabis as a medicine [38–40]. The objective of this review is to synthesise primary research that uses social media discourse and internet search engine queries to answer the following questions:

- In what way, is online user-generated text used as a data source in the investigation of cannabis as a medicine?
- What are the aims, data sources, methods, and research themes of studies using online user-generated text to discuss the medicinal use of cannabis?

## Materials and methods

### Search strategy

For this review, we used an established methodological framework for scoping reviews to inform our methodology and we reported the review in accordance with the PRISMA reporting guidelines [38–41]. Literature database queries were developed for four categories of studies. The first three categories used social media text as a data source, the fourth relied on internet search engine query data. For the first category, the database queries combined words used to describe social media forums, and cannabis-related keywords and general medical-related keywords (Table 1 Category 1). The second category also included the social media and cannabis-related keywords, but used keywords specific to psychiatric disorders, for which the use of medical cannabis has been described. Our search terms for this second category were informed by a systematic review of medicinal cannabis for psychiatric disorders [16] (Table 1 Category 2). The third category included social media and cannabis related keywords but focused on non-psychiatric medical conditions for which cannabis is sometimes used (Table 1 Category 3). The fourth category included studies using Internet search engine queries as a data source, there were no medical conditions included in these searches (Table 1 Category 4). A manual search of MEDLINE: Web of Science (1900–2022), Embase: OVID (1974–2022), Web of Science: Core Collection (1900–2022), and Scopus (1996–2022) databases was conducted by SKH and CMH in May 2021 and again in October 2022. The search was limited to English-language studies that were published between January 1974 and April 2022 (S1 Appendix).

**Table 1:** Search strategy.

**CATEGORY 1—SOCIAL MEDIA, CANNABIS, AND MEDICAL TERMS AS KEYWORDS**		
*Social media related Keywords*	*Cannabis keywords*	*Medical keywords*
‘Social media’ OR twitter OR reddit OR instagram OR youtube OR pinterest OR facebook OR ‘social network forum’ OR ‘Online health community’ OR ‘message board’	cannabis OR cannabis OR cannabinoids OR delta-9-tetrahydrocannabinol OR cannabidiol OR cbd OR cbg OR cbn OR thc OR weed	medical OR medicinal OR patient OR patients OR medicine OR doctor OR position OR care OR therapy OR therapeutic
**CATEGORY 2—SOCIAL MEDIA, CANNABIS, AND PSYCHIATRIC DISORDERS KEYWORDS**		
*Social media related Keywords*	*Cannabis keywords*	*Psychiatric disorders*
‘Social media’ OR twitter OR reddit OR instagram OR youtube OR pinterest OR facebook OR ‘social network forum’ OR ‘Online health community’ OR ‘message board’	cannabis OR cannabis OR cannabinoids OR delta-9-tetrahydrocannabinol OR cannabidiol OR cbd OR cbg OR cbn OR thc OR weed	depression OR depressive OR ‘mental illness*’ OR ‘mental disorder*’ OR ‘mental health’ OR ‘mood disorder*’ OR ‘affective disorder*’ OR anxi* OR ‘panic disorder’ OR ‘obsessive compulsive’ OR adhd OR ‘attention deficit’ OR phobi* OR bipolar OR psychiat* OR psychological OR psychosis OR psychotic OR schizophr* OR ‘severe mental*’ OR ‘serious mental*’ OR antidepress* OR antipsychotic* OR ‘post traumatic*’ OR ‘personality disorder*’ OR stress
**CATEGORY 3—SOCIAL MEDIA, CANNABIS AND VARIOUS MEDICAL (NON-PSYCHIATRIC) CONDITIONS/ ILLNESSES KEYWORDS**		
*Social media related Keywords*	*Cannabis keywords*	*Medical conditions*
‘Social media’ OR twitter OR reddit OR instagram OR youtube OR pinterest OR facebook OR ‘social network forum’ OR ‘Online health community’ OR ‘message board’	cannabis OR cannabis OR cannabinoids OR delta-9-tetrahydrocannabinol OR cannabidiol OR cbd OR cbg OR cbn OR thc OR weed	Pain, Opioid, Alzheimer, sleep, OR insomnia, inflammatory, arthritis, Multiple Sclerosis, Endometriosis
**CATEGORY 4—SEARCH ENGINE QUERIES AND CANNABIS KEYWORDS**		
*Search Engine keywords*	*Cannabis keywords*	
‘Search engine’ OR ‘search log’ OR ‘search queries’ OR ‘online search’ OR ‘internet Search’ OR ‘web search’	cannabis OR Cannabis OR Cannabinoids OR Delta-9-Tetrahydrocannabinol OR Cannabidiol OR CBD OR CBG OR CBN OR THC OR weed	

The inclusion criteria for this review were: (i) peer reviewed research studies, (ii) peer reviewed conference papers (iii) studies which used online user-generated text as a data source, and (iv) social media research that was either directly focused on cannabis and cannabis products that have an impact on health, or were health-related studies that found medicinal use of cannabis.

Exclusion criteria comprised: (i) editorials, letters, commentaries, surveys, protocols and book chapters; (ii) studies that used social media for recruiting participants; (iii) studies where the full text of the publication was not available; (iv) conference abstracts (iv) studies primarily focused on electronic nicotine delivery systems adapted to deliver cannabinoids; (v) studies that used bots or autonomous systems as the main data source and (vi) studies that focused exclusively on synthetic cannabis.

All studies captured by the search queries listed in Table 1 were uploaded into excel to enable all duplicates to be removed. Following this all titles and abstracts were reviewed independently and in duplicate by CMH and SKH. Records were excluded based on title and abstract screening as well as publication type.

The full text articles that were identified for inclusion following screening process were then independently critiqued by pairs of reviewers using a checklist developed for this study. The purpose of the checklist that we developed for this systematic scoping review was to provide an overall assessment of quality rather than generate a specific score, (S2 Appendix). Assessments of quality in each study were based on evidence of relative quality in the aims or objectives, main findings, data collection method, analytic methods, data source, and evaluation and interpretations of the study. CMH and SKH critiqued all articles, and YB and MC each critiqued a selection of studies to ensure each article had been independently reviewed by two researchers. Where initial disagreement existed between reviewers regarding the inclusion of a study, team members met to discuss the disputed article’s status until consensus was achieved.

### Study inclusion

Assessments of quality in each study were based on evaluating each study’s aims and objectives, main findings, data collection and analytic methods, data sources, and evaluation and interpretations of the results. Social media studies were included if there were no major biases affecting the internal, external or construct validity of the study [42]. In doing so, the internal validity of each study was determined by the quality of the data and analytic processes used, the external validity determined by the extent to which the findings can be generalised to other contexts, and the construct validity was ascertained by the extent to which the chosen measurement tool correctly measured what the study aimed to measure (S3 Appendix).

## Results

**Fig 1:**
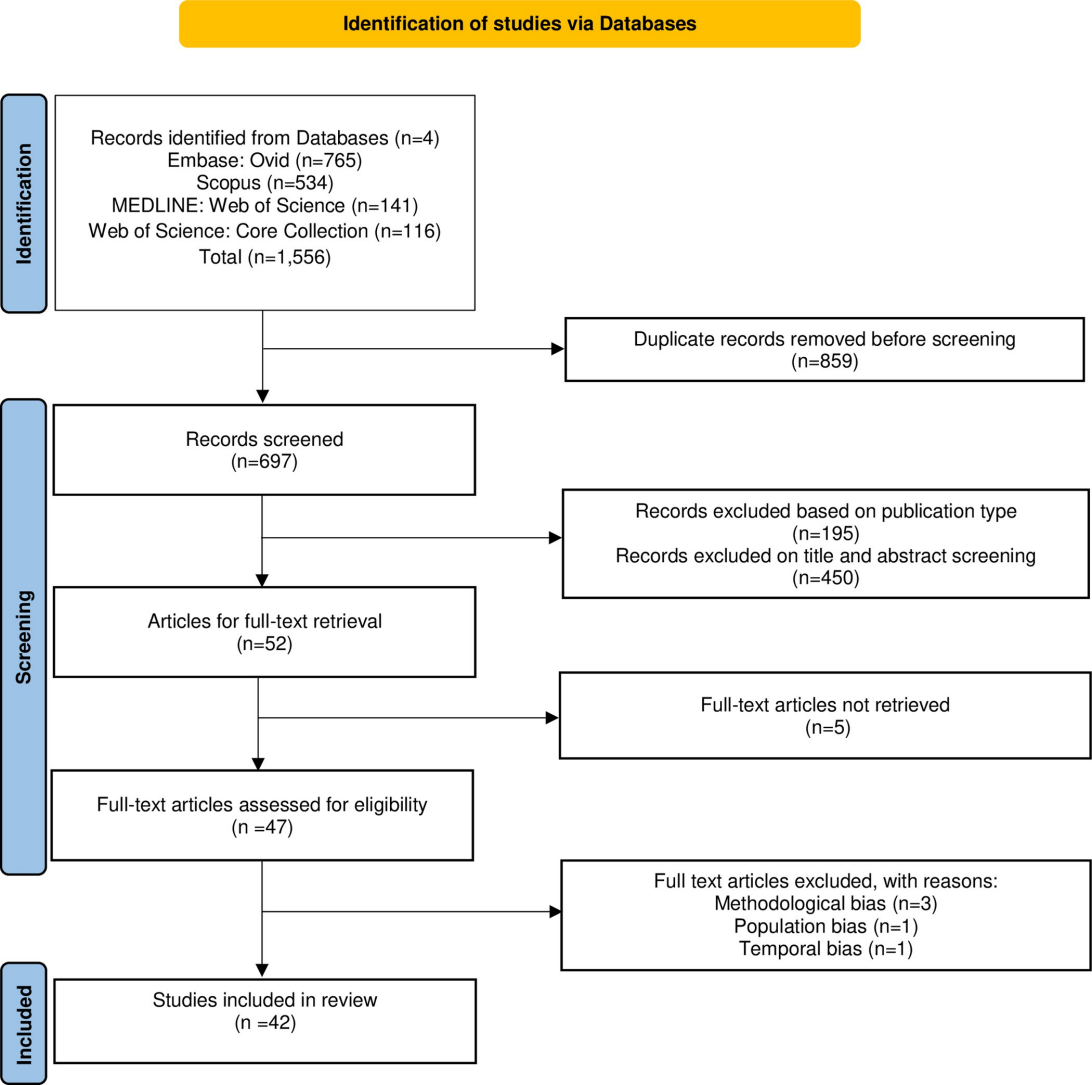
PRISMA flow diagram for a systematic scoping review of social media data and cannabis as a medicine.

Of the 1556 titles identified in the electronic database searches, 859 duplicate articles were removed, 450 were excluded following the screening of title and abstracts and 195 were excluded based on publication type (i.e., survey, letter, comment, abstract). This screening process provided fifty-two potentially relevant full text primary research studies to be included in the review (Fig 1). Of these, five articles were not able to be retrieved, two out of forty-seven articles had initial disagreement. Upon consensus, five were excluded with reasons (S3 Appendix) using the quality assessment checklist as described above. This provided forty-two papers for inclusion in this systematic scoping review published between 2014 and 2022. Regarding publication type, the majority were journal articles 40/42 (95.2%), and two were conference-based publications 2/42 (4.8%). Although the first study was published eight years ago, nearly two-thirds 24/42 (57%), have been published over the last four years. Table 2 provides a summary of each paper that includes author names, publication year, data source, duration of the study, number of collected posts, number of analysed posts, and the coding or labelling approach used.

**Table 2:** Articles included in the review.

#	STUDY	SOURCE	DURATION	COLLECTED DATA	ANNOTATED/ANALYSED	CODING/LABELLING APPROACH
1	McGregor et al., 2014 [44]	Online forums, Facebook, Twitter, YouTube, Patient- Opinion.org.uk	Not Available	3,785 items	All data	Manual coding
2	Cavazos-Rehg, Sowles & Fisher et al., 2015 [100]	Twitter	February 2014—Mar 2014	7,653,738 tweets	7,000 tweets	Manual coding using crowdsourcing services
3	Daniulaityte et al., 2015 [69]	Twitter	October 2014—December 2014	125,255 tweets 27,018 geolocated	All data	No coding—Use existing geographical fields
4	Gonzalez-Estrada et al., 2015 [46]	YouTube	4–8 June 2014	200 most viewed videos	All data	Manual coding
5	Krauss et al., 2015 [47]	YouTube	22 January 2015	116 Videos	All data	Manual coding
6	Thompson, Rivara & Whitehill, 2015 [43]	Twitter	March 2012—July 2013	36,939 original tweets 10,000 retweets	~47,000 tweets	Manual coding
7	Cavazos-Rehg, Sowles & Krauss et al., 2016 [49]	Twitter	January 2015	206,854 tweets	5,000 tweets	Manual coding using crowdsourcing services
8	Lamy et al., 2016 [50]	Twitter	May 2015—July 2015	100,182 tweets 26,975 geolocated	3,000 tweets	Manual coding
9	Mitchell et al., 2016 [51]	Online Forums	October 2014	268 forum threads	46 threads 880 posts	Manual coding
10	Andersson, Persson & Kjellgren, 2017 [52]	Online Forums	18–19 April 2016	32 topics	All data	Manual coding
11	Dai & Hao, 2017 [72]	Twitter	August 2015—April 2016	1,253,872 post traumatic stress disorder (PTSD) tweets 66,000 cannabis PTSD tweets	2,000 labelled tweets, remaining tweets by machine learning	Automated coding using machine learning
12	Greiner, Chatton & Khazaal, 2017 [53]	Online Forums	November 2014—March 2015	717 posts	All data	Manual coding
13	Turner & Kantardzic, 2017 [73]	Twitter	August 2015—April 2016	40,509 geolocated tweets	2,000 labelled, remaining tweets by machine learning	Automated coding using machine learning
14	Westmaas, McDonald & Portier, 2017 [74]	Online Forums	January 2000—December 2013	468,000 posts	All data	Automated coding using topic modelling
15	Yom-Tov & Lev-Ran, 2017 [75]	Bing search engine	November 2016—April 2017	Not available	All data	Automated coding using lexicons
16	Cavazos-Rehg, Krauss & Sowles et al., 2018 [54]	YouTube	10–11 June 2015	83 videos	All data	Manual coding
17	Glowacki, Glowacki, & Wilcox., 2018 [76]	Twitter	August 2016—October 2016	73,235 tweets	All data	Automated coding using topic modelling
18	Meacham, Paul & Ramo, 2018 [77]	Reddit	January 2010–December 2016	~400,000 posts	All data	Automated coding using lexicons and pattern matching
19	Leas, Nobels & Caputi et al., 2019 [66]	Google Trends	January 2004—April 2019	Not available	Summary data	No coding–used Google trends data
20	Meacham, Roh & Chang et al., 2019 [55]	Reddit	January 2017—December 2017	193 dabbing questions and/or posts	All data	Manual coding
21	Nasralah, El-Gayar & Wang, 2019 [70]	Twitter	January 2015—February 2019	20,609 tweets	All data	Automated coding supplied by an analytics company
22	Pérez-Pérez et al., 2019 [83]	Twitter	February 2018—August 2018	24,634 tweets	All data	Automated coding using lexicons
23	Shi et al., 2019 [67]	Google Trends; Buzzsumo	January 2011—July 2018	Not available	Summary data	No coding–used Google trends data
24	Allem, Escobedo & Dharmapuri, 2020 [78]	Twitter	May 2018—December 2018	19,081,081 tweets	60,861 non-bots 8,874 bots	Automated coding using rule-based methods
25	Janmohamed et al., 2020 [85]	Blogs, news articles, forums, comments, professional reviews, Facebook posts	August 2019—April 2021	4,027,172 documents and/or blog entries	All data	Automated coding using topic modelling
26	Jia et al., 2020 [56]	Google, Facebook, YouTube	September 2019	51 Google websites 126 Facebook posts 37 YouTube videos	All data	Manual coding
27	Leas, Hendrickson & Nobels et al., 2020 [57]	Reddit	January 2014—August 2019	104,917 reddit posts	3,000 initial data 376 as testimonials	Manual coding
28	Merten, Gordon & King et al., 2020 [58]	Pinterest	January 2018—December 2018	1,280 pins	226 pins	Manual coding
29	Mullins et al., 2020 [71]	Twitter	June 2017—July 2017	941 tweets	All data	Automated coding supplied by an analytics company
30	Saposnik & Huber, 2020 [68]	Google Trends	January 2004—December 2019	Not available	Summary data	No coding–used Google trends data
31	Song et al., 2020 [59]	GoFundMe	January 2012—December 2019	1,474 campaigns	500 campaigns	Manual coding
32	Tran & Kavuluru, 2020 [84]	Reddit and FDA comments	January 2019—April 2019	64,099 reddit comments 3,832 FDA (U.S. Food & Drug Administration) comments	All data	Automated coding using rule-based methods
33	van Draanen et al., 2020 [79]	Twitter	January 2017—June 2019	1,200,127 tweets	All data	Automated coding using Topic modelling
34	Zenone, Snyder & Caul, 2020 [65]	GoFundMe	January 2017—May 2019	155 campaigns	All data	Manual coding
35	Pang et al., 2021 [62]	Twitter	December 2019—December 2020	17,238 tweets	1,000 tweets	Manual coding
36	Rhidenour et al., 2021 [63]	Reddit	January 2008—December 2018	974 posts	All data	Manual coding
37	Smolev et al., 2021 [64]	Facebook	November 2018—November 2019	7,694 posts	All data	Manual coding
38	Zenone, Snyder & Crooks, 2021 [61]	GoFundMe	June 2017—May 2019	164 campaigns	All data	Manual coding
39	Soleymanpour, Saderholm & Kavuluru, 2021 [80]	Twitter	July 2019	2,200,000 tweets	2,000 labelled, remaining data by machine learning	Automated coding using machine learning
40	Allem et al. 2022 [81]	Twitter	January 2020—September 2020	353,353 tweets	1,092 labelled, remaining data by classifiers	Automated coding using rule-based methods
41	Meacham, Nobles, Tompkins & Thrul, 2022 [60]	Reddit (2 subreddits)	December 2015—August 2019	16,791 opioid recovery subreddits 159,994 opioid use subreddits	200 posts labelled manually and analysed 908 opioid recovery subreddits 4,224 opioid use subreddits	Manual coding and automated coding using rule-based methods
42	Turner, Kantardzic, & Vickers-Smith, 2022 [82]	Twitter		567,850 tweets collected –	5,496 tweets manually labelled 167,755 personal use tweets 143,322 commercial tweets labelled by classifier	Automated coding using machine learning

Adapted from: Page MJ, McKenzie JE, Bossuyt PM, Boutron I, Hoffmann TC, Mulrow CD, et al. The PRISMA 2020 statement: an updated guideline for reporting systematic reviews. BMJ 2021;372:n71. doi: 10.1136/bmj.n71. For more information, visit: http://www.prisma-statement.org/.

### Data collection and annotation

The largest manually annotated dataset that contained 47,000 labelled tweets was published by Thompson et al. in 2015 [43]. This paper was one of 22 studies included in this review (52.4%) that either collected a limited number of data points, or sampled their collected data, and manually coded the data to gain an in-depth understanding of the domain [44–65]. Four of the 42 studies (9.5%) used existing meta-data including Google trends summary data [66–68], and geo-location data [69]. Two (4.8%) studies used data that was manually coded using crowdsourcing services [45,49], and two (4.8%) used automated coding supplied by a social media analytics company [70,71]. Fifteen of the forty-two studies (35.7%) used automated methods for labelling data, which included the use of machine learning, lexicon, and rule-based algorithms [60,72–85]. Automated coding was increasingly used as an analytic tool for social media data on this topic from 2017 onward (Table 2).

### Data analysis

For the studies that were manually labelled, analysis included the calculation of proportions and trends, and the development of repeating and emergent themes. [43,44,46,47,49,50,54–56,60,61,64,65,82,86–94]. Leas, Nobles et al. 2019, Shi, Brant et al. 2019, and Saposnik and Huber 2020, reported on Google trends data that delivered an index of Google search trends over time [66–68]. Daniulaityte, Nahass et al. 2015, processed Twitter data that contained existing geographical fields to identify geolocation at source [69]. The studies that utilised a large volume of data used advanced computational methods, which included sentiment analysis, topic modelling, and rule-based text mining [60,72,77–82,85]. The use of sentiment analysis in the [60,80,95] enabled the analysis of people’s sentiments, opinions, and attitudes. Topic modelling in the [74,76,79,80,85] studies enabled the development of themes via automatic machine learning methods. The use of rule-based text mining such as found in the [60,72,83,96,97] studies enabled the classification of posts into pre-existing health-related categories.

### Research themes

In this review, we categorized the forty-two research articles into six broad themes. Themes were based on the research questions motivating the studies, where each paper was classified as belonging a primary theme, based on alignment with the research aims (Table 3).

**Table 3:** Themes in reviewed articles.

THEME	STUDY	AIM
General cannabis related conversations (n = 9)	Cavazos-Rehg et al., 2015 [100]	Topic analysis of cannabis sentiment
	Thompson, Rivara & Whitehill, 2015 [43]	Content analysis of cannabis-related conversations
	Greiner, Chatton & Khazaal, 2017 [53]	Analysis of self-help online forums related to cannabis use and addiction
	Turner & Kantardzic 2017 [73]	Examination of geographical impact on cannabis conversations; topic analysis, and social networks analysis
	Cavazos-Rehg et al., 2018 [54]	Content analysis of YouTube cannabis reviews and examine exposure to these reviews and their impact on cannabis use.
	Allem, Escobedo & Dharmapuri, 2020 [78]	Topic analysis of cannabis-related Twitter conversations to capture and describe the public’s recent experiences
	Van Draanen et al., 2020 [79]	Topic and sentiment analysis of geographical impact on cannabis conversations;
	Rhidenour et al., 2021 [63]	Exploration of Veterans discussions regarding their cannabis use on Reddit
	Allem et al., 2022 [78]	Content analysis of Twitter for cannabis related motivations and health consequence use
Cannabis mode of use (n = 7)	Daniulaityte et al., 2015 [69]	Thematic analysis of concentrate use (‘dabs’) conversations and examine geographical impact on cannabis
	Krauss et al., 2015 [47]	To explore the content of cannabis dabbing-related videos on YouTube
	Cavazos-Rehg et al., 2016 [49]	Thematic analysis of dabbing conversations to investigate the consequences of dabbing high-potency cannabis
	Lamy et al., 2016 [50]	Thematic analysis of edibles conversations and examine geographical impact on Cannabis
	Meacham, Paul & Ramo, 2018 [77]	Analysis of discussions of emerging and traditional forms of cannabis use
	Meacham et al., 2019 [55]	Thematic analysis of dabbing questions and responses
	Janmohamed et al.,2020 [85]	Topic analysis of vaping narrative before and during COVID-19 pandemic
Cannabis for a specific condition (n = 6)	Mitchell et al., 2016 [51]	Content analysis of posts about cannabis for ADHD
	Dai & Hao, 2017 [72]	Analysis of cannabis use for PTSD and the geographical impacts on cannabis use
	Shi et al., 2019 [67]	Trends in use of cannabis for cancer and analysis of popular cannabis for cancer news
	Jia et al., 2020 [56]	Content analysis of cannabis & glaucoma related posts
	Zenone, Snyder & Caul, 2020 [65]	Content analysis of crowdfunding cancer campaigns
	Pang et al., 2021 [62]	Thematic analysis of pregnancy & cannabis related posts
Cannabis as a treatment option for illness and disease (n = 12)	McGregor et al., 2014 [44]	To analyse the ophthalmic content of social media platforms
	Gonzalez-Estrada et al., 2015 [46]	Content analysis of asthma related YouTube videos
	Andersson, Persson, Kjellgren, 2017 [52]	Thematic analysis of cluster headaches and migraines conversation
	Westmaas, McDonald & Portier, 2017 [74]	Topic analysis of smoking related conversations on a cancer survivor forum
	Glowacki et al.,2018 [76]	Topic modelling of opioid related tweet
	Nasralah, El-Gayar & Wang, 2019 [70]	To understand the concerns of opioid-addicted users
	Pérez-Pérez et al., 2019 [83]	To characterize the bowel disease community on Twitter
	Mullins et al., 2020 [71]	Analysis of common pain discussion topics
	Saposnik & Huber, 2020 [68]	Analysis of trends in web searches for the cause and treatments of autism spectrum disorder
	Song et al., 2020 [59]	Analysis of patient’s perspective for using complementary and alternative medicine or declining conventional cancer therapy
	Smolev et al., 2021 [64]	Thematic analysis of brachial plexus injury Facebook conversation
	Meacham, Nobels et al., 2022 [60]	Analysis of active opioid use subreddit and opioid recovery subreddits
Cannabidiol (CBD) related posts (n = 7)	Merten et al., 2020 [58]	Analysis of how CBD is portrayed on Pinterest
	Zenone, Snyder & Crooks, 2021 [61]	Analysis of CBD information pathways
	Leas et al., 2019 [66]	Analysis of public interest trends in CBD
	Leas et al., 2020 [57]	Analysis of reasons for CBD consumption
	Tran & Kavuluru, 2020 [84]	To examine the remedial effect and modes of use of CBD
	Soleymanpour, Saderholm & Kavuluru, 2021 [80]	Content analysis of marketing claims for CBD in Twitter
	Turner et al 2022 [82]	Content analysis of personal and commercial tweets of cannabidiol related tweets
Adverse drug reactions and adverse effects (n = 1)	Yom-Tov & Lev-Ran, 2017 [75]	Prevalence of internet search engine queries relating to the topic of adverse reactions and cannabis use.

#### General cannabis-related conversations

Nine studies were included in the theme relating to cannabis-related conversations (Table 4). The main keywords used in these studies included general terms such as ‘cannabis’, ‘marijuana’, ‘pot’, and ‘weed’. The major aim of these studies was to either identify topics of conversations regarding cannabis, or to examine the role of normative and valence information in the perception of medicinal cannabis. These studies are included because they reported on conversations around cannabis use for medical purposes, the valence associated with perceptions of health benefits of cannabis, and reports of adverse effects. For example, a study on veterans use of cannabis found that cannabis is used to self-medicate a number of health issues, including Post-Traumatic Stress Disorder (PTSD), anxiety and sleep disorder [94]. Seven of the studies used Twitter as a data source [43,73,78,87,96,98,99], one examined the content of YouTube videos about cannabis [100], one investigated online self-help forums [88] and another used Reddit data [94].

**Table 4:** General cannabis related conversations.

STUDY	AIM	HEALTH-RELATED EFFECTS/CLAIMS	DATA IDENTIFICATION
Cavazos-Rehg, Krauss et al., 2015 [100]	To examine the sentiment and themes of cannabis-related tweets from influential users and to describe the users’ demographics.	A common theme of pro tweets was that cannabis has health benefits. Anti-cannabis posts spoke of the harm experienced in using cannabis. 77% of posts had positive sentiments, with 12 times higher reach than other posts.	Cannabis-related keywords
Thompson, Rivara et al., 2015 [43]	To examine cannabis- related content in Twitter, especially content tweeted by adolescent users, and to examine any differences in message content before and after the legalisation of recreational cannabis in two US states.	More tweets described perceived positive benefits of cannabis use, including relaxation and escaping life problems. Tweets described cannabis as less harmful than other drugs or as not harmful at all and suggested its medical role for conditions such as depression and cancer. Less than 1% of tweets expressed a concern about cannabis use.	Cannabis-related keywords
Greiner, Chatton et al., 2017 [53]	To investigate online content of cannabis use/addiction self-help forums.	Self-help forums on cannabis share a theme around cannabis users seeking help for addiction and withdrawal issues.	
Turner et al., 2017 [73]	To examine if cannabis legalisation policies impact Twitter conversations and the social networks of users contributing to cannabis conversations.	Medical cannabis was a major topic in the conversations.	Cannabis-related keywords
Cavazos-Rehg, Krauss et al., 2018 [54]	To investigate cannabis product reviews and the relationship between exposure to product reviews and cannabis users’ demographics and characteristics.	Product reviews promoted cannabis for helping with relaxation, pain relief, sleep, improving emotional well-being. Medical cannabis users are more likely to be exposed to cannabis product reviews.	
Allem, Escobedo et al., 2020 [78]	To identify and describe cannabis-related topics of conversation on Twitter, and the public health implications of these.	Health and medicine were the third most prevalent topic of the 12 topics identified in the data. Posts suggested that cannabis could help with cancer, sleep, pain, anxiety, depression, trauma, and posttraumatic stress disorder. Health-related posts from social bots were almost double that of genuine posts.	Cannabis-related keywords
Van Draanen et al., 2020 [79]	To examine differences in the sentiment and content of cannabis-related tweets in the US (by state cannabis laws) and Canada.	Medical cannabis use was one of the main topics of conversations in cannabis-related tweets from both countries.	Tweets filtered on US and Canada geolocation and then further filtered on cannabis-related keywords
Rhidenour et al., 2021 [63]	To explore Veterans’ Reddit discussions regarding their cannabis use.	Over a third of the Reddit posts described the use of medical cannabis as an aid for psychological and physical ailments. Overall, veterans discussed how the use of medical cannabis reduced PTSD symptoms, anxiety, and helped with their sleep.	The veteran subreddit
Allem, Majmundar et al., 2022 [81]	To determine the extent to which a medical dictionary could identify cannabis-related motivations for use and health consequences of cannabis use.	There were posts related to both health motivations and consequences of cannabis use. The health-related posts included issues with the respiratory system, stress to the immune system, and gastrointestinal issues, among others.	Cannabis-related keywords

#### Cannabis mode of use

Seven studies were included in the theme relating to the mode of use of cannabis as a medicine (Table 5). These studies collected data using keywords such as ‘vape,’ ‘vaping’, ‘dabbing’, and ‘edibles’. Conversations around modes of use revealed a theme about lacking, seeking, or sharing knowledge about health consequences of the modes of use. Another theme was around the perceived health benefits of cannabis and the various modes of use of cannabinoids that included sleep improvement and relaxation resulting from dabbing oils [49] or consuming ‘edibles’ [50]. The findings suggest that for emerging modes of use such as dabbing, where the availability of evidence-based information is limited, people seek information from others’ experiences.

**Table 5:** Cannabis mode of use.

STUDY	AIM	HEALTH-RELATED EFFECTS/CLAIMS	DATA IDENTIFICATION
Daniulaityte et al., 2015 [69]	To explore Twitter data on concentrate (‘dabs’) use and examine the impact of cannabis legalisation policies on concentrate use conversations.	Twitter data suggest popularity of dabs in the US states with legalised recreational/medical use of cannabis. Dabbing as an emerging mode of use could carry significant health risks.	Dab-related keywords for U.S. location
Krauss, Sowles et al., 2015 [47]	To explore the content of cannabis dabbing-related videos on YouTube.	Only 21% of videos contained warnings about dabbing, such as preventing explosions, injury, or negative side effects. 22% of videos specifically mentioned medical cannabis or getting ‘medicated’, either in the video itself or in the accompanying text description.	Dabbing related keywords
Cavazos-Rehg, Sowles et al., 2016 [49]	To study themes of dabbing conversations and to investigate the consequences of high-potency cannabis consumption.	The fourth theme (of seven) was about cannabis helping with relaxation, sleep or solving problems. Extreme effects were both physiological and psychological. The most common physiologic effects were passing out and respiratory, with coughing the most common respiratory effect.	Dabbing related keywords
Lamy, Daniulaityte et al., 2016 [50]	To study themes of edibles conversations and examine legalisation policies’ impact on cannabis-related tweeting activity.	Twitter data suggest mostly positive attitudes toward cannabis edibles. Positive tweets describe the quality of the ‘high’ experienced and how cannabis edibles facilitate falling asleep. Negative tweets discuss the unreliability of edibles’ THC dosage and delayed effects that were linked to over-consumption, which could lead to potential harmful consequences.	Cannabis edible-related keywords
Meacham, Paul et al., 2018 [77]	To analyse discussions of emerging and traditional forms of cannabis use.	Less than 2% of conversations described adverse effects. The most mentioned adverse effects were anxiety-related in the context of smoking, edibles, and butane hash oil, and ‘cough’ for vaping and dabbing.	A cannabis specific subreddit on various modes of use
Meacham, Roh et al., 2019 [55]	To study themes of dabbing- related questions and responses.	Health concerns are the fifth category of dabbing questions—including respiratory effects, anxiety, and vomiting. Respondents in these conversations usually spoke from personal experience.	Search for ‘Dab’ and ‘question’ on cannabis subreddits
Janmohamed et al.,2020 [85]	To map temporal trends in the web-based vaping narrative, to indicate how the narrative changed from before to during the COVID-19 pandemic.	The emergence of a vape-administered CBD treatment narrative around the COVID-19 pandemic.	Vape-related keywords

#### Cannabis as a medicine for a specific health issue

Six studies were included in the theme relating to cannabis as a medicine for a specific health issue (Table 6). These studies investigated conversations around the use of cannabis or cannabidiol (CBD) for a specific health issue. The health conditions included glaucoma [56], PTSD [72], cancer [65,101], Attention Deficit Hyperactivity Disorder (ADHD) [91], and pregnancy [92]. These studies mostly discovered that conversations claimed benefits of cannabis as an alternative treatment for these health conditions, although mentions of harm, and both harm and therapeutic effects, were also present [91].

**Table 6:** Cannabis as a medicine for a specific health issue.

STUDY	AIM	HEALTH-RELATED EFFECTS/CLAIMS	DATA IDENTIFICATION
Mitchell et al., 2016 [51]	To examine the content of online forum threads on ADHD and cannabis use to identify trends about their relation, particularly regarding therapeutic and adverse effects of cannabis on ADHD.	Of all individual posts, 25% indicated that cannabis is therapeutic for ADHD, as opposed to 8% that claim it is harmful, 5% that it is both therapeutic and harmful, and 2% that it has no effect on ADHD.	Cannabis and ADHD keywords
Dai & Hao, 2017 [72]	To evaluate factors that could impact public attitudes to PTSD related cannabis use.	Of all PTSD tweets, 5.3% were related to cannabis use and these tweets predominantly supported cannabis use for PTSD.	Cannabis and PTSD keywords
Shi et al., 2019 [67]	To characterize trends in use of cannabis for cancer and analysis of content and impact of popular news about cannabis for cancer.	Between 2011–2018, the relative google search volume of ’cannabis cancer’ queries increased at a rate ten times faster than ’standard cancer therapies’ queries. Popular ‘false news’ stories had a much higher engagement than contrary ‘accurate’ news stories.	Cannabis vs standard therapies for cancer
Jia, Mehran et al., 2020 [56]	To analyze the content quality and risk of readily available online information regarding cannabis and glaucoma.	While the American Glaucoma Society recommends against cannabis use for glaucoma treatment, 21% of Facebook, 24% of Google, and 59% of YouTube search results were pro cannabis use for glaucoma treatment.	Cannabis and Glaucoma keywords
Zenone et al., 2020 [65]	To use crowdfunding campaigns to understand how cannabidiol is represented/misrepresented as a cancer-related care.	CBD use was reported to reduce the side effects of conventional treatments or can be used with other complementary cancer treatments. Reported uses included stimulating appetite, general pain relief, assisting with sleep, countering nausea, or general recovery purposes. Most campaigners presented definite efficacy of CBD for pain or symptom management.	CBD term variants and ‘cancer’
Pang et al., 2021 [62]	To examine cannabis and pregnancy-related tweets over a 12-month period.	Thirty-six percent mentioned safety during pregnancy, 2.3% of posts asked about safety during postpartum, and 2.7% of posts expressed use of cannabis during pregnancy to help with pregnancy symptom i.e., to help with morning sickness, nausea, vomiting, headaches, pain, stress, and fatigue. The authors conclude that health providers discuss risks and provide official information about cannabis use in pregnancy.	Cannabis and pregnancy related keywords

#### Cannabis as a medicine as part of discourse on illness and disease

Twelve studies were included in the theme relating to cannabis as a medicine as part of discourse on illness and disease (Table 7). In this theme, the research focus was on social media topics relating to management and treatment options for a range of health conditions rather than on medicinal cannabis per se. Health conditions discussed included inflammatory and irritable bowel disease [102], opioid use disorder [70,103,104], pain [71], ophthalmic disease [44], cluster headache and migraine [86], asthma [105], cancer [93,106], autism disorder [107], and brachial plexus injury [64].

**Table 7:** Cannabis as a medicine as part of discourse on illness and disease.

STUDY	AIM	HEALTH-RELATED EFFECTS/CLAIMS	DATA IDENTIFICATION
McGregor et al., 2014 [44]	To analyse the ophthalmic content of social media platforms.	Treatment was one of the main themes, with complementary therapy featuring most prominently on Twitter, where 87% of posts on complementary therapy described the use of medical cannabis for glaucoma.	Glaucoma patient forums and glaucoma keywords
Gonzalez-Estrada et al., 2015 [46]	To determine the educational quality of YouTube videos for asthma.	The most common video content was regarding alternative medicine (38%) and included cannabis as well as live fish ingestion; salt inhalers; raw food, vegan, gluten-free diets; yoga; Ayurveda; reflexology; acupressure; and acupuncture; and Buteyko breathing.	Asthma related videos
Andersson et al., 2017 [52]	To understand the use of non-established or alternative pharmacological treatments used to alleviate cluster headaches and migraines.	Cannabis was discussed for its potential to alleviate symptoms or reduce the frequency of migraine attacks. Some discussed use of cannabis for other purposes, but experienced additional benefits for headache symptoms. The effects of self-treatment with cannabis appeared more contradictory and complex than treatment with other substances.	Search for ‘treatment migraines’ on three alternative treatment online forums
Westmaas et al., 2017 [74]	To investigate contexts in which smoking, or quitting is discussed in a cancer survivor network.	Use of cannabis (primarily for nausea), was the fourth topic.	Smoking/cessation-related keywords from the Cancer Survivors Network (CSN)
Glowacki et al.,2018 [76]	To identify public reactions to the opioid epidemic by identifying the most popular topics.	Mentions of cannabis as an effective alternate to opioids for managing pain.	Opioid-related keywords
Nasralah et al., 2019 [70]	To understand the concerns of opioid-addicted users.	Cannabis was found in two of five main themes: ‘In recovery’ and ‘taking illicit drugs’ for pain management.	Users who self-identified as addicted to, or previously addicted to, opioids
Pérez-Pérez et al., 2019 [83]	To characterize the bowel disease community on Twitter.	Medical cannabis was the fourth most mentioned term in the bowel disease (BD) community. Medical cannabis and its components were the most discussed drug, with mentions of its benefits in mitigating common BD symptoms.	Inflammatory bowel disease, Irritable bowel disease keywords
Mullins et al., 2020 [71]	To examine pain-related tweets in Ireland over a 2-week period.	The fourth most occurring keyword was cannabis. Ninety percent of cannabis related tweets were non-personal, with highly positive sentiment and highest number of impressions per tweet. Cannabis had by the largest number of tweets aimed at generating awareness.	Pain-related keywords
Saposnik & Huber, 2020 [68]	To analyse of trends in web searches for the cause and treatments of autism spectrum disorder (ASD).	ASD and cannabis web searches have continued to rise since 2009. Apart from searches on Applied Behavioral Analysis and Autism, cannabis and ASD have been searched more than other ASD interventions since 2013.	‘Autism’ and key search terms for causes and treatments of autism
Song et al., 2020 [59]	To understand the cancer patient’s perspective for using complementary and alternative medicine, or for declining traditional cancer therapy.	Cannabidiol oil was 10th amongst the most used alternative treatments.	Twenty most prevalent cancers in the U.S. and a list of most frequently utilised complementary and alternative medicine including yoga, herbal, and meditation.
Smolev et al., 2021 [64]	To analyse themes of brachial plexus injury Facebook conversations.	There were 313 posts regarding cannabinoids as a preferred alternative pain management medication.	‘traumatic brachial plexus injury’ keyword
Meacham, Nobles et al. 2022 [60]	To assess and compare active opioid use subreddit and an opioid recovery subreddit: 1) the proportion of posts that mention cannabis, 2) the most frequently-used words and phrases in posts that mention cannabis, and 3) motivations for cannabis use in relation to opioid use as described in cannabis-related posts.	All posts (title and content) and associated metadata posted from December 2015 to August 2019 (45 months) on an opioid use subreddit and an opioid recovery subreddit were downloaded from the Pushshift Reddit database [34] via Google Big Query. Data were analyzed in R Studio version 1.2.5019. Duplicate posts were removed (1.5% of posts), which were identified with the ‘duplicated’ base R function applied to post text.	weed, cannabis, marijuana, pot, reefer, ganja, thc, and cbd

#### Cannabidiol (CBD)

There were seven studies in the cannabidiol category [61,80,82,89,90,108,109] (Table 8). These studies concentrated on conversations related to the benefits of CBD products, product sentiment (positive, negative, or neutral), the factors that impact on a person’s decision to use CBD products, and the trends in therapeutic use of CBD.

**Table 8:** Cannabidiol (CBD) related posts.

STUDY	AIM	HEALTH-RELATED EFFECTS/CLAIMS	DATA IDENTIFICATION
Merten, Gordon et al., 2020 [58]	To analyse how CBD is portrayed on Pinterest.	Most pins (57.5%) did not make a specific health benefit claim yet 42.5% claimed mental, physical, or both mental and physical health benefits.	‘cannabidiol’ or ‘CBD’
Zenone, Snyder et al., 2021 [61]	To analyse the CBD informational pathways which bring consumers to CBD for medical purposes.	Self-directed research was the most common pathway to CBD. The proposed uses of CBD were for cancer, seizure-inducing diseases/conditions, joint/inflammatory diseases, mental health disorders, nervous system diseases, and autoimmune diseases.	‘cannabidiol’ or ‘CBD’
Leas, Nobel et al., 2019 [66]	To analyse public interest trends in CBD using Google Trends.	Searches for CBD exceed searches for yoga and around half as much as searches for dieting.	‘cannabidiol’ or ‘CBD’ vs other alternative medicine including diet, yoga,
Leas, Hendrickson et al., 2020 [57]	To assess if individuals are using CBD for diagnosable conditions which have evidence-based therapies.	Psychiatric conditions were the most cited diagnosable condition, mentioned in 63.9% of testimonials. The second most cited subcategory was orthopedic conditions (26.4%), followed by sleep (14.6%), neurological (6.9%), and gastroenterological (3.9%) conditions.	CBD subreddit posts
Tran & Kavuluru, 2020 [84]	To examine social media data to determine perceived remedial effects and usage patterns for CBD.	Anxiety disorders and pain were the two conditions dominating much of the discussion surrounding CBD, both in terms of general discussion and for CBD as a perceived therapeutic treatment. CBD is mentioned as a treatment for mental issues (anxiety, depression, stress) and physiological issues (pain, inflammation, headache, sleep disorder, seizure disorders, nausea, and cancer).	CBD subreddit posts
Soleymanpour et al., 2021 [80]	To perform content analysis of marketing claims for CBD in Twitter.	Over 50% of CBD tweets appear to be marketing related chatter. Pain and anxiety are the most popular conditions mentioned in marketing messages. Edibles are the most popular product type being advertised, followed by oils.	‘cbd’, ‘cbdoil’, and ‘cannabidiol’
Turner, Kantardzic et al. 2021 [82]	The objective of this study was to provide a framework for public health and medical researchers to use for identifying and analyzing the consumption and marketing of unregulated substances using CBD as an exemplar.	There was a significant difference in the sentiment scores between the personal and commercial CBD tweets, the mean sentiment score of the commercial CBD scores was higher than that of the personal CBD score. Pain, anxiety, and sleep had the highest positive sentiment score for both personal and commercial CBD tweets.	‘CBD’, ‘cannabidiol.’

#### Adverse drug reactions and adverse effects

One paper had a research question that explicitly focused on the detection of adverse events [75] (Table 9) This study explored the prevalence of internet search engine queries relating to the topic of adverse reactions and cannabis use. Seven other studies contained mentions of adverse effects which were associated with cannabis use [47,49,51,53,55,62,77,81], however these papers were not included under this theme, as their research questions were not centered around the explicit investigation of adverse events.

**Table 9:** Cannabis related to adverse drug reactions and adverse effects.

STUDY	AIM	HEALTH-RELATED EFFECTS/CLAIMS	DATA IDENTIFICATION
Yom-Tov & Lev-Ran, 2017 [75]	To check if search engine queries can be used to detect adverse reactions of cannabis use.	A high correlation between the side effects recorded on established reporting systems and those found in the search engine queries. These side effects included anxiety, depression-related symptoms, psychotic symptoms such as paranoia and hallucinations, cough, and other symptoms.	Cannabis related keywords

## Discussion

Currently, there exist systematic reviews of cannabis and cannabinoids for medical use based on clinical efficacy outcomes from randomised controlled trials [18] and reviews on the use of social media for illicit drug surveillance [110]. However, following searches on PROSPERO and the databases listed above, to our knowledge, this paper constitutes the first systematic scoping review examining studies that used user-generated online text to understand the use of cannabis as a medicine in the global community.

Our scoping review found that the use of social media and internet search queries to investigate cannabis as a medicine is a rapidly emerging area of research. Over half of the studies included in this review were published within the last four years (24, 57.1%), this reflects not only increase community interest in the therapeutic potential of cannabinoids, but also world-wide trends towards cannabis legalisation [4,7–9,111,112]. Regarding social media platforms, Twitter was the data source in eighteen (42.9%) of the forty-two studies, almost three and a half times the number of studies using Reddit (6, 14.3%) and just under three times the number of studies using data from Online forums (5, 11.9%). Three (7.14%) GoFundMe studies and three (7.14%) Google Trends studies were also included in the review. Hence, much of the data in this systematic review comprised posts from the Twitter platform. Several factors may explain this finding, firstly Twitter is real-time in nature, it has a high volume of messages, and it is publicly accessible. These factors makes it a useful data source for public health surveillance [113].

Regarding the subjects of the studies, twelve (28.6%) focused on general user-generated content regarding the treatment of health conditions (glaucoma, autism, asthma, cancer, bowel disease, brachial plexus injury, cluster headaches, opioid disorder). These studies were either explicitly designed to investigate cannabis as a medicine or were studies that generated results that incidentally found cannabis mentioned as an alternative or complementary treatment (either formally prescribed or via self-medication).

Qualitative studies featured in the research, but while their contributions are valuable, especially in the context of hypothesis generation, they tend to be limited by their smaller datasets, which frequently comprise manually annotated samples. The recent emergence of powerful machine learning-based natural language processing (NLP) models suggests that it should be possible to automate the continuous processing of far larger datasets using NLP technologies, built upon the insights gained from initial qualitative studies, and even leveraging their annotated data for training purposes. Recent trends in the social science data landscape have shown a convergence between social science and computer science expertise, where the ability to use computational methods has greatly assisted the collection and validation of robust datasets that can form the basis of deeper social science research [114].

We found much heterogeneity in approaches applied to analyse user-related content, and inconsistent quality in the methodologies adopted. While we endeavored to include as many studies as possible, some of the publications initially identified as suitable for inclusion were not suitable based on a minimum quality requirements checklist (S1 Appendix). This checklist was designed to ensure that selection of data source, choice of platform, data acquisition and preparation, analysis and evaluation delivers data and conclusions that are appropriate for answering the research questions.

The utilisation of user-generated content for health research is subject to several inherent limitations which include; the lack of control that researchers have in relation to the credibility of information, the frequently unknown demographic characteristics and geographical location of individuals generating content, and the fact that social media users are not necessarily representative of the wider community [115]. Furthermore, the uniqueness, volume, and salience of social media data has implications that need to be considered when used for health information analysis [116]. Volume is usually inversely related to salience; a platform such as Twitter has a very high volume of information, much of which is not highly pertinent for the analysis of an effect, whereas the information contained in a blog will contain less volume, but will be more salient for analysis. Notwithstanding these limitations, user-generated content comprises large-scale data that provides access to the unprompted organic opinions and attitudes of cannabis users in their own words and is an effective medium through which to gauge public sentiment. To date, insights regarding cannabis as medicine have gained primarily through surveys or focus groups which have their own limitations regarding the format of data collection and potential bias in participant recruitment. A limitation of this scoping review was the lack of inclusion of a computational database such as *IEEE Xplore* in the search strategy, and the exclusion of the search terms ‘infodemiology’ and ‘infoveillance.’ Infodemiology and infoveillance studies explicitly use web-based data for research, and *IEEE Xplore* is a repository that contains technical papers and documents relating to computer science. However, our search was systematic, comprehensive and *IEEE Xplore* is Scopus-indexed, and we expect data loss to be minimal.

## Conclusion

Our systematic scoping review reflects a growing interest in the use of user-generated content for public health surveillance. It also demonstrates there is a need for the development of a systematic approach for evaluating the quality of social media studies and highlights the utility of automatic processing and computational methods (machine learning technologies) for large social media datasets. This systematic scoping review has shown that user-generated content as a data source for studying cannabis as a medicine provides another means to understand how cannabis is perceived and used in the community. As such, it is another potential ‘tool’ with which to engage in pharmacovigilance of, not only cannabis as a medicine, but also other novel therapeutics as they enter the market.

## Supporting information

S1 Checklist (DOCX)

S1 Appendix Search strategies for each database. (DOCX)

S2 Appendix Quality assessment checklist. (DOCX)

S3 Appendix Excluded studies. (DOCX)

S1 Database (DOCX)

S2 Database (XLSX)
