# Supplementary material for: Social media discourse and internet search queries on cannabis as a medicine: A systematic scoping review
Source: PLoS One. 2023 Jan 20;18(1):e0269143. doi: 10.1371/journal.pone.0269143 (PMC9858862; doi:10.1371/journal.pone.0269143)
Supplement: S2 Appendix — (DOCX) [file pone.0269143.s003.docx]

**Quality Assessment Checklist**

| SECTION/TOPIC | CHECKLIST ITEMS SUMMARY STATEMENT | PRIMARY RESEARCH | | | |
| --- | --- | --- | --- | --- | --- |
|  |  | Reviewer 1 | | Reviewer 2 | |
|  |  | Yes/No/Na | Notes | Yes/No/Na | Notes |
| Research context | The basic research requirements including abstract, background, and research questions exist and are well-explained. |  |  |  |  |
| Data collection | The selection of data source, search keywords and data acquisition methods deliver appropriate data for the research. |  |  |  |  |
| Data pre-processing/curation | Data cleaning, handling of missing data and enrichment of data is done and is valid and reliable. |  |  |  |  |
| Study Methodology | Methodology used for the study is suitable and creates valid results. |  |  |  |  |
| Data Analysis | The analysis includes descriptions of the goals and justifications of the methods used. |  |  |  |  |
| Evaluation and Interpretation | Evaluation metrics are explained. Biases are accounted for and interpretations are consistent with results. |  |  |  |  |
| Other criteria | Study includes ethical considerations and funding resources and conflicts of interest are clear. |  |  |  |  |
