# Supplementary material for: Social media discourse and internet search queries on cannabis as a medicine: A systematic scoping review
Source: PLoS One. 2023 Jan 20;18(1):e0269143. doi: 10.1371/journal.pone.0269143 (PMC9858862; doi:10.1371/journal.pone.0269143)
Supplement: S3 Appendix — (DOCX) [file pone.0269143.s004.docx]

| EXCLUDED STUDIES | | | |
| --- | --- | --- | --- |
| 1 | Manveer Mann, Whitney Ginder & Sang-Eun Byun (2022) | Highs and Lows of Cannabis Decriminalization: Twitter Analysis and Ethical and Regulatory Implications for Retailing and Marketing | The study has temporal biases as the data is collected for one day in response to the house vote. Bias affecting study external validity and generalisability. |
| 2 | Mamo, Szeto, Mirhossaini, Fortugno & Dellavalle (2021) | Tetrahydrocannabinol and Skin Cancer: Analysis of YouTube Videos | Small sample size of 10 You-tube videos introducing data bias and impacting sample representativeness. Analysed using subjective measure of overall quality Discern & GQS score |
| 3 | Wang et al (2020) | Consumer interest in topical cannabidiol (CBD) An examination of online search trends from 2015-2019 | Poorly described analysis/methods impacting internal and external validity of the study |
| 4 | Narayhanan et al. (2020) | Cannabidiol (CBD) Oil, Cancer, and Symptom Management A Google Trends Analysis of Public Interest | Not clearly defined research question, bias in the study methodology and interpretation of results |
| 5 | Li, Kakani & Li (2020) | Understanding cannabis information on social media: Examining tweets from verified, regular, and suspended users | Methodological limitations in the qualitative component of study. |
