# Supplementary material for: Social media discourse and internet search queries on cannabis as a medicine: A systematic scoping review
Source: PLoS One. 2023 Jan 20;18(1):e0269143. doi: 10.1371/journal.pone.0269143 (PMC9858862; doi:10.1371/journal.pone.0269143)
Supplement: S1 Database — (DOCX) [file pone.0269143.s005.docx]

| CATEGORY 1 | SOCIAL MEDIA, CANNABIS, AND MEDICAL TERMS AS KEYWORDS | | |
| --- | --- | --- | --- |
| Database | Web of Science (Core Collection) | Scopus | Embase (OVID) |
| Date Searched | 19 September 2022 | 19 September 2022 | 19 September 2022 |
| Query | TS=(cannabis OR Marijuana OR Cannabinoids OR Delta-9-Tetrahydrocannabinol OR Cannabidiol OR CBD OR CBG OR CBN OR thc OR weed)  AND TS=("Social media" OR "online forum" OR "internet forum" OR Twitter OR Reddit OR Instagram OR YouTube OR Pinterest OR Facebook OR "social network forum" OR "Online health community" OR "message board") AND TS=(medical OR medicinal OR patient OR patients OR medicine OR Doctor OR position OR care OR therapy OR therapeutic) | (TITLE-ABS-KEY (cannabis AND cannabis OR marijuana OR cannabinoids OR delta-9-tetrahydrocannabinol OR cannabidiol OR cbd OR cbg OR cbn OR thc OR weed) AND TITLE-ABS-KEY ("Social media" OR twitter OR reddit OR instagram OR youtube OR pinterest OR facebook OR "social network forum" OR "Online health community" OR "message board") AND TITLE-ABS KEY (medical OR medicinal OR patient OR patients OR medicine OR doctor OR position OR care OR therapy OR therapeutic) AND LANGUAGE (english)) AND PUBYEAR > 1973 AND PUBYEAR < 2023 | ((cannabis or Marijuana or Cannabinoids or Delta-9-Tetrahydrocannabinol or Cannabidiol or CBD or CBG or CBN or THC or weed) and ("Social media" or Twitter or Reddit or Instagram or YouTube or Pinterest or Facebook or "online forum" or "internet forum" or "social network forum" or "Online health community" or "message board") and (medical or medicinal or patient or patients or medicine or Doctor or position or care or therapy or therapeutic)).mp. [mp=title, abstract, heading word, drug trade name, original title, device manufacturer, drug manufacturer, device trade name, keyword heading word, floating subheading word, candidate term word] limit 1 to (human and english language) |
| Date Filter | 01 January 1974 - 01 April 2022 | 01 January 1996 - 01 April 2022 | 01 January 1974 - 01 April 2022 |
| Results = 638 | 116 titles retrieved | 214 titles retrieved | 308 titles retrieved |
| CATEGORY 2 | SOCIAL MEDIA, CANNABIS, AND PSYCHIATRIC DISORDERS KEYWORDS | | |
| Database | MEDLINE (Web of Science) | Scopus | Embase (OVID) |
| Date Searched | 19 September 2022 | 19 September 2022 | 19 September 2022 |
| Query | TS = (cannabis OR Marijuana OR Cannabinoids OR Delta-9-Tetrahydrocannabinol OR Cannabidiol OR CBD OR CBG OR CBN OR thc OR weed) AND TS = (depression or depressive or mental illness* or mental disorder* or mental health or mood disorder* or affective disorder* or anxi* or panic disorder or obsessive compulsive or adhd or attention deficit or phobi* or bipolar or psychiat* or psychological or psychosis or psychotic or schizophr* severe mental* or serious mental* or antidepress* or antipsychotic* or post traumatic* or personality disorder* or stress) AND TS = ("Social media" OR "online forum" OR "internet forum" OR Twitter OR Reddit OR Instagram OR YouTube OR Pinterest OR Facebook OR "social network forum" OR "Online health community" OR "message board") | TITLE-ABS-KEY ((cannabis OR marijuana OR cannabinoids OR delta-9-tetrahydrocannabinol OR cannabidiol OR cbd OR cbg OR cbn OR thc OR weed) AND (depression OR depressive OR "mental illness*" OR "mental disorder*" OR "mental health" OR "mood disorder*" OR "affective disorder*" OR anxi* OR "panic disorder" OR "obsessive compulsive" OR adhd OR "attention deficit" OR phobi* OR bipolar OR psychiat* OR psychological OR psychosis OR psychotic OR schizophr* OR "severe mental*" OR "serious mental*" OR antidepress* OR antipsychotic* OR "post traumatic*" OR "personality disorder*" OR stress) AND ("Social media" OR "online forum" OR "internet forum" OR twitter OR reddit OR instagram OR youtube OR pinterest OR facebook OR "social network forum" OR "Online health community" OR "message board")) AND (LIMIT-TO (DOCTYP, "ar") OR LIMIT-TO (DOCTYPE, "cp")) | (cannabis OR Marijuana OR Cannabinoids OR Delta-9-Tetrahydrocannabinol OR Cannabidiol OR CBD OR CBG OR CBN OR thc OR weed) AND (depression or depressive or "mental illness*" or "mental disorder*" or "mental health" or "mood disorder*" or "affective disorder*" or anxi* or "panic disorder" or "obsessive compulsive" or adhd or "attention deficit" or phobi* or bipolar or psychiat* or psychological or psychosis or psychotic or schizophr* or "severe mental*" or "serious mental*" or antidepress* or antipsychotic* or "post traumatic*" or "personality disorder*" or stress) AND ("Social media" OR "online forum" OR "internet forum" OR Twitter OR Reddit OR Instagram OR YouTube OR Pinterest OR Facebook OR "social network forum" OR "Online health community" OR "message board") |
| Date Filter | 01 January 1974 - 01 April 2022 | 01 January 1996 - 01 April 2022 | 01 January 1974 - 01 April 2022 |
| Results = 421 | 65 titles retrieved | 116 titles retrieved | 240 titles retrieved |
| CATEGORY 3 | PAIN, CANNABIS AND SOCIAL MEDIA | | |
| Database | MEDLINE (Web of Science) | Scopus | Embase (OVID) |
| Date Searched | 19 September 2022 | 19 September 2022 | 19 September 2022 |
| Query | TS = (cannabis OR Marijuana OR Cannabinoids OR Delta-9-Tetrahydrocannabinol OR Cannabidiol OR CBD OR CBG OR CBN OR thc OR weed) AND TS = pain AND TS = ("Social media" OR "online forum" OR "internet forum" OR Twitter OR Reddit OR Instagram OR YouTube OR Pinterest OR Facebook OR "social network forum" OR "Online health community" OR "message board")) | TITLE-ABS-KEY ((cannabis OR marijuana OR cannabinoids OR delta-9-tetrahydrocannabinol OR cannabidiol OR cbd OR cbg OR cbn OR thc OR weed) AND ("Social media" OR twitter OR reddit OR instagram OR youtube OR pinterest OR facebook OR "social network forum" OR "Online health community" OR "message board") AND pain) | ((cannabis or Marijuana or Cannabinoids or Delta-9-Tetrahydrocannabinol or Cannabidiol or CBD or CBG or CBN or thc or weed) and ("Social media" or Twitter or Reddit or Instagram or YouTube or Pinterest or Facebook or "online forum" or " internet forum" or "social network forum" or "Online health community" or "message board") and pain).mp. [mp=title, abstract, heading word, drug trade name, original title, device manufacturer, drug manufacturer, device trade name, keyword heading word, floating subheading word, candidate term word] |
| Date Filter | 01 January 1974 - 01 April 2022 | 01 January 1996 - 01 April 2022 | 01 January 1974 - 01 April 2022 |
| Results = 162 | 26 titles retrieved | 52 titles retrieved | 84 titles retrieved |
| CATEGORY 4 | SEARCH ENGINE QUERIES AND CANNABIS KEYWORDS | | |
| Database | MEDLINE (Web of Science) | Scopus | Embase (OVID) |
| Date Searched | 23 September 2022 | 23 September 2022 | 23 September 2022 |
| Query | TS= (cannabis OR marijuana OR cannabinoids OR delta-9-tetrahydrocannabinol OR cannabidiol OR cbd OR cbg OR cbn OR thc OR weed) AND ("Search engine" OR "search log" OR "search queries" OR "online search" OR "internet Search" OR "web search") | TITLE-ABS-KEY ((cannabis OR marijuana OR cannabinoids OR delta-9-tetrahydrocannabinol OR cannabidiol OR cbd OR cbg OR cbn OR thc OR weed) AND ("Search engine" OR "search log" OR "search queries" OR "online search" OR "internet Search" OR "web search")) | ((cannabis OR marijuana OR cannabinoids OR delta-9-tetrahydrocannabinol OR cannabidiol OR cbd OR cbg OR cbn OR thc OR weed) AND ("Search engine" OR "search log" OR "search queries" OR "online search" OR "internet Search" OR "web search")) .mp. [mp=title, abstract, heading word, drug trade name, original title, device manufacturer, drug manufacturer, device trade name, keyword heading word, floating subheading word, candidate term word] |
| Date Filter | 01 January 1974 - 01 April 2022 | 01 January 1996 - 01 April 2022 | 01 January 1974 - 01 April 2022 |
| Results = 335 | 50 titles retrieved | 152 titles retrieved | 133 titles retrieved |
